# Comparative Transcriptome Analysis Revealed Genes Involved in Sexual and Polyploid Growth Dimorphisms in Loach (*Misgurnus anguillicaudatus*)

**DOI:** 10.3390/biology10090935

**Published:** 2021-09-18

**Authors:** Li-Fei Luo, Zi-Sheng Xu, Eman Abdelwareth Baioumy Elsayed Elgazzar, Hang Du, Dan-Yang Li, Xiao-Yun Zhou, Ze-Xia Gao

**Affiliations:** 1 Key Lab of Freshwater Animal Breeding, Ministry of Agriculture, Key Lab of Agricultural Animal Genetics, Breeding and Reproduction of Ministry of Education, Engineering Research Center of Green Development for Conventional Aquatic Biological Industry in the Yangtze River Economic Belt, Ministry of Education, College of Fisheries, Huazhong Agricultural University, No. 1 Shizishan Street, Hongshan District, Wuhan 430070, China; luolifei@mail.hzau.edu.cn (L.-F.L.); xu-zisheng@webmail.hzau.edu.cn (Z.-S.X.); xiaoruDong@webmail.hzau.edu.cn (E.A.B.E.E.); hbdh1996@163.com (H.D.); lidanyang20210727@163.com (D.-Y.L.); 2 Hubei Hongshan Laboratory, Wuhan 430070, China; 3 Engineering Technology Research Center for Fish Breeding and Culture in Hubei Province, Wuhan 430070, China

**Keywords:** *Misgurnus anguillicaudatus*, sexual size dimorphism, polyploid size dimorphism, growth, comparative transcriptome, gene expression

## Abstract

**Simple Summary:**

*Misgurnus anguillicaudatus* not only exhibits sexual size dimorphism, but also shows polyploid size dimorphism. Here, we performed comparative transcriptome integration analysis of multiple tissues of diploid and tetraploid *M. anguillicaudatus* of both sexes. We found that differences in energy metabolism and steroid hormone synthesis levels may be the main causes of sexual and polyploidy growth dimorphisms of *M. anguillicaudatus*. Fast-growing *M. anguillicaudatus* (tetraploids, females) have higher levels of energy metabolism and lower steroid hormone synthesis and fatty acid degradation abilities than slow-growing *M. anguillicaudatus* (diploids, males).

**Abstract:**

Sexual and polyploidy size dimorphisms are widespread phenomena in fish, but the molecular mechanisms remain unclear. Loach (*Misgurnus anguillicaudatus*) displays both sexual and polyploid growth dimorphism phenomena, and are therefore ideal models to study these two phenomena. In this study, RNA-seq was used for the first time to explore the differentially expressed genes (DEGs) between both sexes of diploid and tetraploid loaches in four tissues (brain, gonad, liver, and muscle). Results showed that 21,003, 17, and 1 DEGs were identified in gonad, liver, and muscle tissues, respectively, between females and males in both diploids and tetraploids. Regarding the ploidy levels, 4956, 1496, 2187, and 1726 DEGs were identified in the brain, gonad, liver, and muscle tissues, respectively, between tetraploids and diploids of the same sex. When both sexual and polyploid size dimorphisms were considered simultaneously in the four tissues, only 424 DEGs were found in the gonads, indicating that these gonadal DEGs may play an important regulatory role in regulating sexual and polyploid size dimorphisms. Regardless of the sex or ploidy comparison, the significant DEGs involved in glycolysis/gluconeogenesis and oxidative phosphorylation pathways were upregulated in faster-growing individuals, while steroid hormone biosynthesis-related genes and fatty acid degradation and elongation-related genes were downregulated. This suggests that fast-growing loaches (tetraploids, females) have higher energy metabolism levels and lower steroid hormone synthesis and fatty acid degradation abilities than slow-growing loaches (diploids, males). Our findings provide an archive for future systematic research on fish sexual and polyploid dimorphisms.

## 1. Introduction

Sexual size dimorphism, which is the relative difference in body size and growth rate between males and females of the same species, has been observed in many cultivable fish species, and this phenomenon varies widely across species. Some species, such as carp (*Cyprinus carpio*), rainbow trout (*Oncorhynchus mykiss*), Japanese flounder (*Paralichthys olivaceus*), and half-smooth tongue sole (*Cynoglossus semilaevis*), show sexual size dimorphism, with females being larger and growing faster than males. In contrast, some other species, such as Nile tilapia (*Oreochromis niloticus*), yellow catfish (*Pelteobagrus fulvidraco*), and channel catfish (*Ictalurus punctatus*), show that males have a faster growth rate and larger body size than females [1]. It is often assumed that the growth difference is mediated by differences in the expression of genes present in both sexes [2,3]. For example, *growth hormone* (*gh*) expression level of females was higher than that of males in *C. semilaevis* and spotted scat (*Scatophagus argus linnaeus*), in which females grow faster than males [4,5]. The sex phenotype is established by several genes that act together to form a complicated regulatory network [6]. Comparative transcriptome analysis can provide a basis for exploring the genetic and molecular mechanisms of sexual dimorphism. With the rapid development of next-generation sequencing (NGS) technologies, RNA-seq technology has become an attractive, low-cost, and highly accurate method that has revealed an increasing number of novel transcripts and sequence variations [7,8], and has also been used to analyze important trait-related gene pathways in organisms, even those without genomic information. Recently, RNA-Seq has been used to identify sex-biased genes in many fish species, such as platy fish (*Xiphophorus maculatus*) [9], *O. mykiss* [10], *O. niloticus* [11], rockfish (*Sebastiscus marmoratus*) [12], catfish (*Ictalurus* *punctatus*) [13], and turbot (*Scophthalmus maximus*) [14]. Such studies have provided some overview of sex-biased gene expression in fish and offer more useful information about sexual dimorphism.

Some fish not only exhibit sexual size dimorphism, but also show polyploid size dimorphism. Polyploids tend to be larger in size and grow faster than diploids in multiple fish species. For example, polyploids in the genus *Barbus* and the Japanese spined loach (*Cobitis biwae*) are larger than those in diploids [15]. Polyploidy in fish is also associated with improved longevity and better ecological adaptability compared to diploids [16]. Currently, most studies on different ploidy individuals in animals have focused on biological characteristics [17], genes related to sex differentiation [18], and comparison of genomic methylation [19]. There are only a few studies on polyploid growth dimorphism in fish, mainly focusing on the differential expression of growth axis-related genes and their regulatory hormone genes in fishes with different ploidy. By comparing the expression of GH/IGF axis genes of *Carassius* carp with different ploidy, it has been revealed that elevated expression of GH/IGF axis genes in triploids plays a crucial role in the faster growth rate of triploids [20]. Tao et al. carried out studies on cDNA cloning and expression of the *cyp19a1a* gene in *Carassius* carp with different ploidy [21]. Polyploids are generally known to have higher levels of genetic diversity than diploid species, owing to the larger total number of chromosome copies [22]. Although there have been some reports on the transcriptome of different ploidy fish [23,24], the molecular regulatory mechanisms of the growth difference between different ploidy fish are still unclear.

Individual growth traits in fish are an important economic characteristic, and is closely linked with fish farming production in aquatic products. In addition to being regulated by the well-known “hypothalamus-pituitary-liver” axis, the growth of vertebrates is also regulated by several energy metabolic processes, such as glycolysis/gluconeogenesis and oxidative phosphorylation. Glycolysis/gluconeogenesis plays an important role in biological synthesis and catabolism [25]. The main physiological functions of glycolysis and gluconeogenesis are the breakdown and synthesis of sugars, respectively. They are two major metabolic pathways that provide precursors and rapid energy sources for cell growth [26]. In Drosophila and zebrafish, glycolysis is located downstream of the GH/IGF axis and plays an important regulatory role in muscle development and animal growth [27]. Oxidative phosphorylation, like glycolysis, is the main source of cellular energy [28]. Oxidative phosphorylation not only supports the growth of tumor cells, but also plays an important role in the growth of normal differentiated somite cells [29,30]. In animals, glucose homeostasis is mutually controlled by catabolic glycolysis/oxidative phosphorylation and anabolic gluconeogenesis pathways [31], thus providing energy for growth.

To date, studies on the regulation of sexual and polyploid growth dimorphisms-related genes in fish are limited. Loach (*Misgurnus anguillicaudatus*), a small freshwater fish species widely distributed in eastern Asia, including China, Korea and Japan, possesses both sexual and polyploid growth dimorphisms [32]. In loach, females grow faster than males and tetraploids grow faster than diploids; therefore, this species is a suitable model to clarify whether fish sexual and polyploid growth dimorphisms are regulated through the same signaling pathways and genes. In this study, comparative transcriptome profiling of brain, gonad, liver, and muscle tissues of 18-month-old diploid and tetraploid males and females was performed to identify the DEGs related to the growth difference between male and female individuals of the same ploidy and between different ploidies of the same sex. Through comparative transcriptome integration analysis of multiple tissues of diploids and tetraploids of both sexes, the key genes and pathways that regulate sexual and polyploid growth dimorphisms of loach are revealed. Our study provides basic information for breeding loach strains with fast growth and uniform size.

## 2. Materials and Methods

### 2.1. Ethic Statement

All experimental protocols were approved by the Animal Experimental Ethical Inspection of Laboratory Animal Center, Huazhong Agricultural University, Wuhan, China (HZAUDO-2016-005, 2016-10-26). All surgery was performed under MS-222 (Sigma, Saint Louis, MO, USA; 100 mg/L) anesthesia, and all efforts were made to minimize suffering. All experiments were performed in accordance with relevant guidelines and regulations.

### 2.2. Sampling and RNA Extraction

Loaches for sampling were collected from the Fisheries Experimental Station of Huazhong Agricultural University. A total of 100 loaches were separately selected from previously established 18-month-old diploid and tetraploid loach populations. The ploidy of fish was determined by flow cytometry analysis as previously described [33], and *Paramisgurnus dabryanus*, which is a known diploid, was used as a control (Figure S1). Forty-eight healthy loaches (24 diploids and 24 tetraploids, containing both females and males) were randomly selected and acclimatized in two tanks with adequate aeration at 24–26 °C for one week before processing. Four tissues (brain, gonad, liver and muscle) of female and male of diploid and tetraploid individuals were immediately sampled separately. Total RNA from each sample was isolated using RNAiso Plus (TaKaRa, Dalian, China) according to the manufacturer’s instructions. RNA quality and concentration were determined using an Agilent Bioanalyzer 2100 (Agilent Technologies, Santa Clara, CA, USA) and Qubit^®^ RNA Assay Kit in Qubit^®^ 2.0 Fluorometer (Life Technologies, Carlsbad, CA, USA), respectively. High-quality RNA was subjected to further libraries construction.

### 2.3. Library Construction and Sequencing

Equal amounts of RNA from four individual samples were mixed as one sequencing sample. A total of 1.5 µg RNA per sample was used as input material for the RNA sample preparations following the removal of possible genomic DNA. Sequencing libraries were generated using NEBNext^®^ Ultra™ RNA Library Prep Kit for Illumina^®^ (NEB, Ipswich, MA, USA) following the manufacturer’s recommendations and index codes were added to attribute sequences to each sample. Subsequently, the library preparations were sequenced on an Illumina HiSeq 2500 platform and 150 bp paired-end reads were generated. Library construction and sequencing were performed by Novogene (Beijing, China). In total, 48 sequencing libraries were constructed and transcriptome data were obtained in this study. All sequencing data were uploaded to the Sequence Read Archive (SRA) of the National Center for Biotechnology Information (NCBI accession number PRJNA678824).

### 2.4. Preliminary Analysis of the Transcriptome Data

Raw data (raw reads) in fastq format were first processed using in-house Perl scripts. At this step, clean data (clean reads) were obtained after filtering out reads with adapters and poly-N, as well as low-quality reads (<Q20) from the raw data. Simultaneously, Q20, Q30, GC-content and sequence duplication level of the clean data were calculated.

All subsequent analyses were based on high-quality clean data. The clean reads from the high-quality clean data were then de novo assembled using Trinity software with min_kmer_cov set to 2 as default and all other parameters set to default [34]. The expression level of each transcript was calculated by the expected number of fragments per kilobase of transcript sequence per million base pairs sequenced (FPKM) method [35].

### 2.5. Screening and Functional Analysis of Differentially Expressed Genes (DEGs)

Differential expression analysis of the samples was performed using DEGseq2 [36]. Genes with an adjusted *p*-value (padj) < 0.05 and |log_2_(fold-change)| > 1 were set as the threshold for significantly differential expression.

Gene ontology (GO) enrichment analysis of the differentially expressed genes (DEGs) was implemented in the GOseq R packages based on Wallenius non-central hyper-geometric distribution, which can adjust for gene length bias in DEGs. In GO enrichment analysis, only categories with a corrected *p*-value < 0.05 were considered as enriched in the network. The Kyoto Encyclopedia of Genes and Genomes (KEGG) pathway analysis was used to determine the location of the DEGs in the different pathways. In this study, KOBAS software was used to test the statistical enrichment of DEGs in the KEGG pathways [37]. Pathways with a corrected *p*-value < 0.05 were considered as the enriched items.

### 2.6. Validation of RNA-Seq Data by Quantitative Real-Time PCR

**Figure 1:**
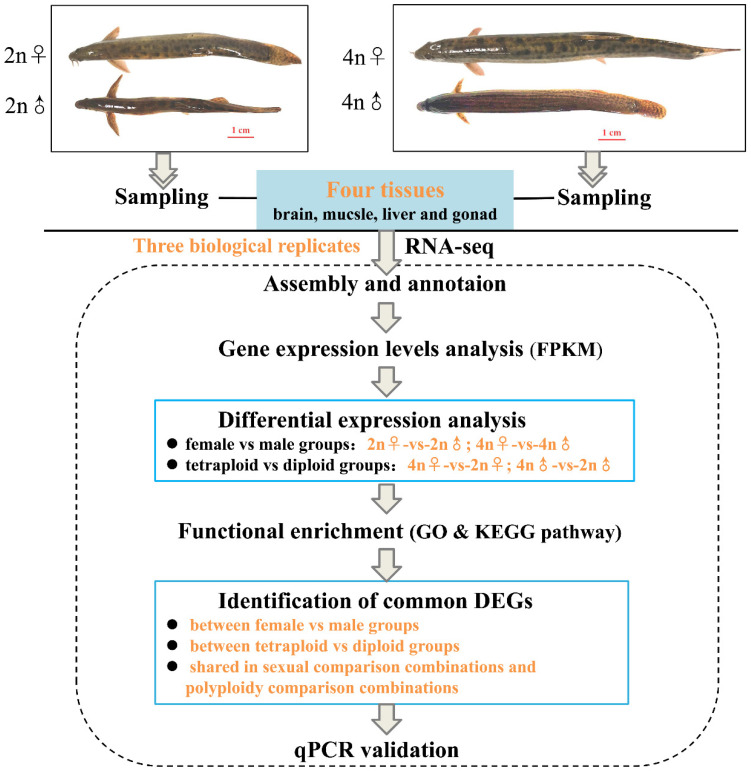
Flow chart of transcriptome sequencing analysis.

To confirm the reliability of the data obtained with RNA-Seq, four genes (*pgk*, *gpi*, *ldh*, and *eno*) involved in the glycolytic pathway, three estrogen receptor genes (*erα*, *erβ1*, and *erβ2*), and several reported growth-related genes (*gh*, *igf1*, *igf2*, *igf1r*, and *igf2r*) were selected for validation by quantitative real-time PCR (qPCR) using the primers listed in Table S1. qPCR was performed on the Applied Biosystems QuantStudio 6 Flex Real-time PCR System (Applied Biosystems, Carlsbad, CA, USA) with SYBR^®^ Premix DimerEraser™ (TaKaRa, Shiga, Japan) according to the manufacturer’s instructions. Five biological replicates were performed, with *β-actin* serving as the reference for internal standardization. The PCR cycling conditions were as follows: 95 °C for 30 s, followed by 40 cycles of 95 °C for 5 s, 60 °C for 30 s, and 72 °C for 30 s. The qPCR results were analyzed using the 2^−ΔΔCt^ method [38]. All data were expressed as mean ± standard deviation (S.D.) values of five replicates. Statistical significance was determined using one-way analysis of variance (ANOVA) followed by multiple comparison testing with the least significant distance (LSD) *t*-test using SPSS 17.0 software. Statistical significance was set at *p*-value < 0.05. The entire research scheme diagram is shown in Figure 1.

## 3. Results

### 3.1. De novo Transcriptome Assembly and Quality Evaluation

**Figure 2:**
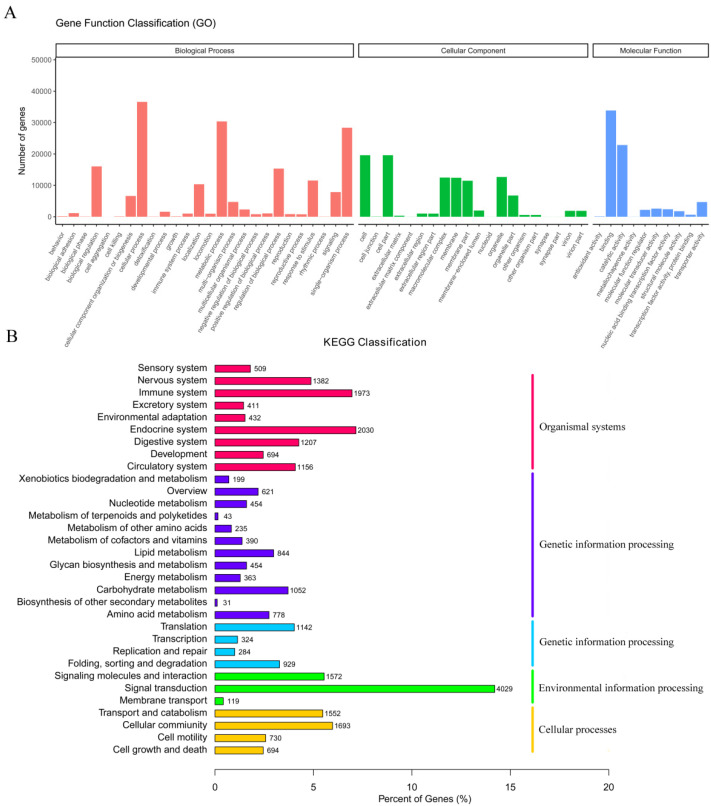
(**A**) GO categorization of the unigenes in the transcriptome of *M. anguillicaudatus.* (**B**) KEGG assignment of unigenes in the transcriptome of *M. anguillicaudatus*.

Before de novo transcriptome assembly, paired-end raw reads were processed to remove adapter fragments and low-quality bases, generating clean reads from raw reads. As shown in Table S2, the raw reads, clean reads, clean bases, Q20, Q30, and the GC content were recorded for 48 libraries. For all libraries, Q20 ≥ 90%, and Q30 ≥ 89%, showing the high-quality of the sequencing data and, thus, could be used for subsequent analyses. After using the Trinity software, de novo sequence assembly resulted in 351,696 unigenes with 956 bp N50 and an average length of 791 bp ranging from 301 bp to 42,939 bp (Tables S3 and S4). The number of unigenes and their length distribution indicated that 68,606 unigenes were >1000 bp in length (Table S3). The functions of unigenes were annotated using GO and KEGG databases. According to GO annotations, the unigenes were involved in 25 biological processes, 16 cell components, and 9 molecular functions (Figure 2A). These unigenes participated in 32 KEGG pathways (Figure 2B).

### 3.2. Analysis of DEGs between Female and Male Loaches

**Figure 3:**
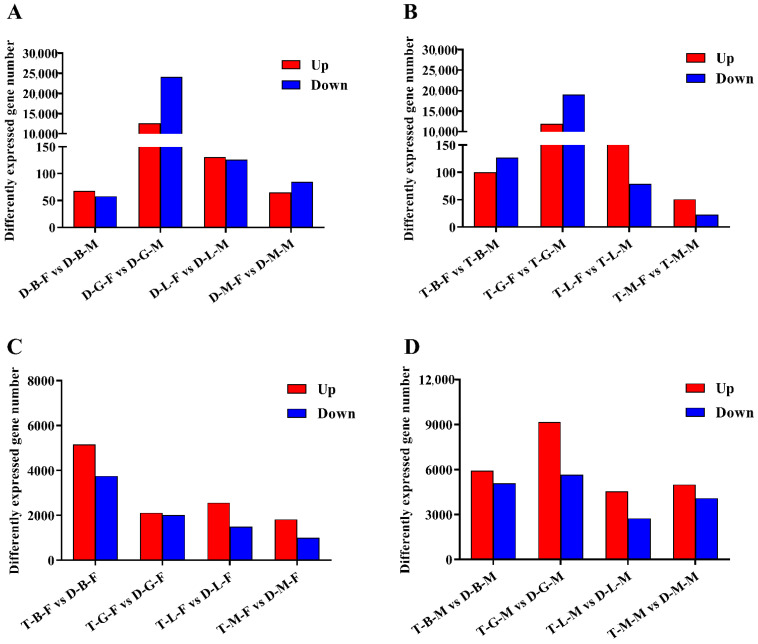
The number of DEGs in four tissues identified in the comparisons of: (**A**) diploid females and diploid males; (**B**) tetraploid females and tetraploid males; (**C**) tetraploid females and diploid females; and (**D**) tetraploid males and diploid males. The letters B, G, L, and M in the middle of each group represent brain, gonad, liver, and muscle, respectively.

Genes were considered DEGs if they showed a padj value < 0.05 and |log_2_(fold-change)| > 1. Among the comparisons of diploid female and male loaches (brain, gonad, liver, and muscle samples from diploid females were compared with those from diploid males, comparisons were named D-B-F vs. D-B-M, D-G-F vs. D-G-M, D-L-F vs. D-L-M, and D-M-F vs. D-M-M), 126 (68 upregulated and 58 downregulated), 36,765 (12,622 upregulated and 24,143 downregulated), 257 (131 upregulated and 126 downregulated), and 150 (65 upregulated and 85 downregulated) DEGs were identified in brain, gonad, liver, and muscle tissues, respectively (Figure 3A). Based on the results of GO annotation, the DEGs in the brain were enriched in three molecular function items, including oxygen binding, heme binding, and tetrapyrrole binding (Table S5). In gonads, 305, 101, and 125 DEGs were enriched in biological processes, cell components, and molecular functions, respectively (Table S6), among which most of the DEGs were involved in protein binding, ion binding, oxidoreductase activity, protein-disulfide reductase activity, and cellular protein modification processes. In the liver, DEGs were significantly enriched in two biological processes and two cell components, which were mainly involved in lipid transport, lipid localization and substrate characteristic transport activity. The DEGs in the muscle were mainly enriched on four molecular functional related items, which were associated with oxidoreductase activity, phosphorylase activity, and glycogen phosphorylase activity (Table S5). Furthermore, according to the KEGG pathway analysis results, the DEGs identified in the brain were mainly involved in steroid hormone biosynthesis and a series of metabolic processes including porphyrin and chlorophyll, amino acids and arachidonic acid metabolism. Meanwhile, the DEGs in gonad, liver, and muscle were mainly associated with cell cycle and PI3K-Akt signaling pathway, phagosome and antigen processing and presentation, glycolysis/gluconeogenesis and HIF-1 signaling pathway, respectively (Table S7).

Among the comparisons of tetraploid female and male loaches (brain, gonad, liver and muscle samples from tetraploid females were compared with those from tetraploid males, and comparisons were named T-B-F vs. T-B-M, T-G-F vs. T-G-M, T-L-F vs. T-L-M, and T-M-F vs. T-M-M), a total of 227 (100 upregulated and 127 downregulated), 30,970 (11,896 upregulated and 19,074 downregulated), 253 (174 upregulated and 79 downregulated), and 74 (51 upregulated and 23 downregulated) DEGs were identified in brain, gonad, liver, and muscle tissues, respectively (Figure 3B). Further GO annotation results showed that there were no GO items in the brain. In gonads, 216, 82, and 116 DEGs were enriched in biological processes, cell components, and molecular functions, respectively (Table S8), among which most of the DEGs were involved in protein binding, ion binding, and oxidoreductase activity (Table S5). In the liver, similar to the GO items of DEGs identified in the D-L-F vs. D-L-M group, the DEGs identified in the T-L-F vs. T-L-M group were also mainly involved in lipid transport, lipid location and matrix characteristic transport activity. In the muscle, unlike the enriched items of DEGs identified in the D-M-F vs. D-M-M group, the DEGs identified in the T-M-F vs. T-M-M group were enriched in the biological processes related to cell cycle and the cell component related to skeletal muscle. The KEGG pathways mainly enriched in the brain, gonad, liver, and muscle were involved in some metabolic processes (protein digestion and absorption, taurine and hypotaurine metabolism), protein processing in endoplasmic reticulum, biosynthesis, and tight junction, respectively (Table S7).

### 3.3. Analysis of DEGs between Tetraploid and Diploid Loaches

To characterize the differences between tetraploid and diploid loaches, the transcriptional levels of the brain, gonad, liver, and muscle tissues of tetraploids and diploids of the same sex were compared. Among the comparisons of tetraploid and diploid female loaches (brain, gonad, liver, and muscle samples from tetraploid females were compared with those from diploid females, and comparisons were named T-B-F vs. D-B-F, T-G-F vs. D-G-F, T-L-F vs. D-L-F, and T-M-F vs. D-M-F, respectively), a total of 8913 (5156 upregulated and 3757 downregulated), 4136 (2114 upregulated and 2022 downregulated), 4069 (2563 upregulated and 1506 downregulated) and 2832 (1820 upregulated and 1012 downregulated) DEGs were identified in brain, gonad, liver, and muscle tissues, respectively (Figure 3C). Among the comparisons of tetraploid and diploid male loaches (T-B-M vs. D-B-M, T-G-M vs. D-G-M, T-L-M vs. D-L-M, and T-M-M vs. D-M-M represent the comparison of brain, gonad, liver, and muscle samples between tetraploid and diploid males, respectively), a total of 11,014 (5931 upregulated and 5083 downregulated), 14,868 (9173 upregulated and 5675 downregulated), 7307 (4559 upregulated and 2748 downregulated), and 9090 (5000 upregulated and 4090 downregulated) DEGs were identified in the brain, gonad, liver, and muscle tissues, respectively (Figure 3D).

GO annotation of the DEGs among the comparisons of tetraploids and diploids of both sexes indicated that the DEGs identified in the brain were associated with DNA integration, DNA metabolic process and oxidoreductase activity. In addition, the DEGs identified in the gonad mainly performed the molecular function of nickel cation binding; those in the liver participated in DNA integration, binding and metabolic processes. Meanwhile, the DEGs in the muscle were enriched in terms of cell components, among which microtubule and actin cytoskeleton were the most represented (Table S5). KEGG pathway analysis further indicated that DEGs between the tetraploids and diploids of both sexes were mainly involved in pathways related to a series of metabolic processes in the brain, cell cycle and oxidative phosphorylation in the gonad, and related to some bacterial infections and their phagocytosis in the liver (Table S7).

### 3.4. Identification of Common DEGs Shared in Different Comparison Groups in the Same Tissue

**Figure 4:**
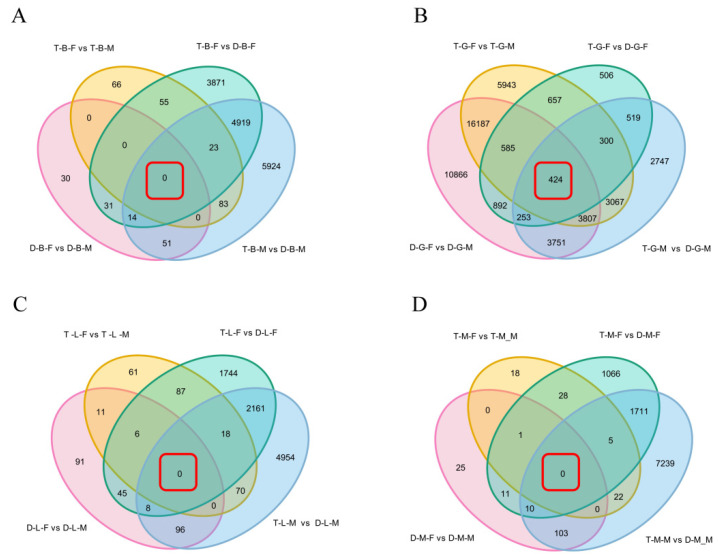
Venn diagram of shared and unique DEGs in brain (**A**), gonad (**B**), liver (**C**), and muscle (**D**) of four comparison groups. The red box indicates the number of DEGs overlapping among the four comparison groups.

In order to obtain the common DEGs shared in different comparison combinations within the same tissue, the DEGs identified in four types of comparative combinations (diploid females vs. diploid males (D-B/G/L/M-F vs. D-B/G/L/M-M), tetraploid females vs. tetraploid males (T-B/G/L/M-F vs. T-B/G/L/M-M), tetraploid females vs. diploid females (T-B/G/L/M-F vs. D-B/G/L/M-F), and tetraploid males vs. diploid males (T-B/G/L/M-M vs. D-B/G/L/M-M) were comprehensively analyzed. When considering the common DEGs between D-B/G/L/M-F vs. D-B/G/L/M-M and T-B/G/L/M-F vs. T-B/G/L/M-M comparison combinations, Venn diagram showed that there were 21,003, 17, and 1 DEGs in the gonad, liver, and muscle, respectively (Figure 4), which were only related to sexes but not ploidy. Surprisingly, there were no common DEGs shared in the comparison combinations of brain (D-B-F vs. D-B-M and T-B-F vs. T-B-M). In addition, the overlapping DEGs in the comparison combinations of T-B/G/L/M-F vs. D-B/G/L/M-F and T-B/G/L/M-M vs. D-B/G/L/M-M were also identified. As shown in Figure 4, there were 4956, 1496, 2187, and 1726 common DEGs shared in the brain, gonad, liver, and muscle, respectively, which were only related to ploidy but not sex. According to KEGG enrichment result, whether it was only related to ploidy or only related to sexes, the DEGs were mainly enriched in the oxidative phosphorylation pathway, metabolic pathways, and cardiac muscle contraction process, among which most of the genes, such as cytochrome c oxidase subunit 2 (*cox2*), *cox3*, *nd4*, *nd5*, and ubiquinol-cytochrome c reductase cytochrome b (*cytb*), were upregulated genes, whereas *cox1* was the most enriched downregulated gene.

Moreover, the overlapping DEGs in the four comparison combinations (D-B/G/L/M-F vs. D-B/G/L/M-M, T-B/G/L/M-F vs. T-B/G/L/M-M, T-B/G/L/M-F vs. D-B/G/L/M-F, and T-B/G/L/M-M vs. D-B/G/L/M-M) were genes related to sexual and polyploidy size dimorphisms of loaches. The Venn diagram showed that there were no common DEGs in the four comparison combinations of brain, liver, and muscle tissues, but there were 424 common DEGs in gonad tissue. KEGG pathway enrichment analysis showed that the common DEGs of different comparison combinations of gonads were mainly involved in transcriptional misregulation in cancer, EGFR tyrosine kinase inhibitor resistance, ErbB signaling pathway, endocrine resistance, and phospholipase D signaling pathway (Table S9), which were associated with a series of cellular processes, including growth, proliferation, and differentiation.

### 3.5. Analysis of KEGG Pathways Related to Sexual and Polyploid Growth Dimorphisms of Loaches

**Figure 5:**
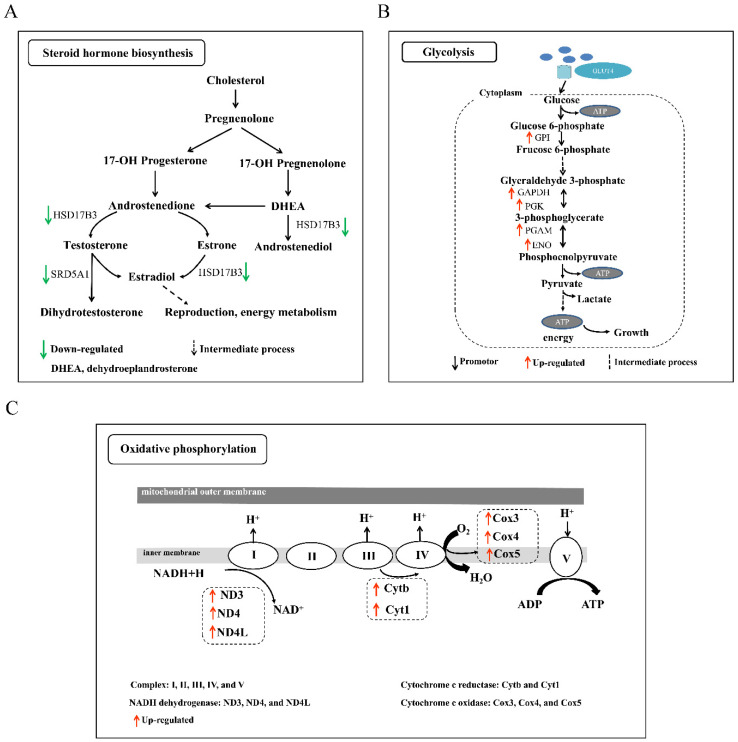
The schematic diagram of key pathways. (**A**) Steroid hormone biosynthesis. Note: HSD17B3, hydroxysteroid 17-beta dehydrogenase 3; SRD5A1, 3-oxo-5-alpha-steroid 4-dehydrogenase 1; DHEA, dehydroepiandrosterone. (**B**) Glycolysis. Note: GPI, glucose-6-phosphate isomerase; GAPDH, glyceraldehyde 3-phosphate dehydrogenase; PGK, phosphoglycerate kinase; PGAM, 2,3-bisphosph-oglycerate-dependent phosphoglycerate mutase; ENO, enolase. (**C**) Oxidative phosphorylation pathway. Note: I, II, III, IV, and V represent different complexes. ND3/4/4L, NADH-ubiquinone oxidoreductase chain 3/4/4L; Cytb/1, ubiquinol-cytochrome c reductase cytochrome b/1; Cox3/4/5, cytochrome c oxidase subunit 3/4/5. The green arrow represents the downregulated, and the dotted arrow represents the intermediate process. The black and red arrow represents the promotor and upregulated DEGs, respectively.

**Figure 6:**
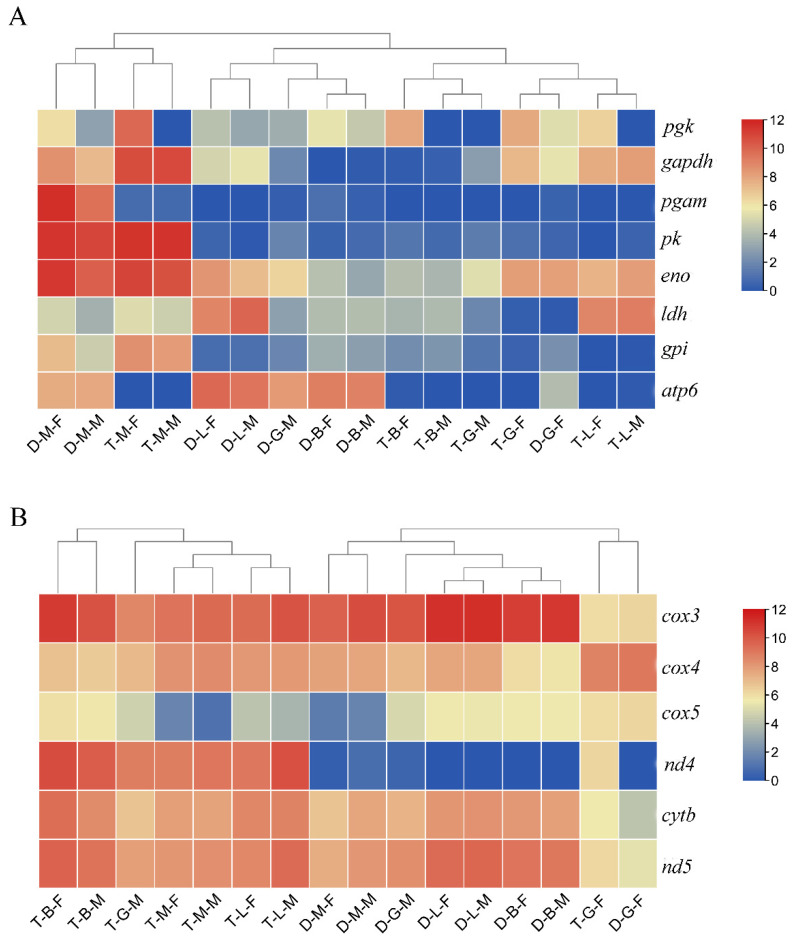
Heat-maps of the key genes related to growth regulation in *M. anguillicaudatus.* (**A**) The heat-map of the key genes related to glycolysis in each group. (**B**) The heat-map of the key genes related to oxidative phosphorylation pathway in each group.

In order to understand the pathways involved in the regulation of sexual growth dimorphism, we focused on the growth-related pathways enriched by DEGs in two types of comparative combinations (D-B/G/L/M-F vs. D-B/G/L/M-M and T-B/G/L/M-F vs. T-B/G/L/M-M). Among the comparisons of diploid females and males (D-B/G/L/M-F vs. D-B/G/L/M-M), the growth-related pathways enriched by DEGs in the brain were steroid hormone biosynthesis and a series of metabolic processes including porphyrin and chlorophyll, amino acids and arachidonic acid metabolism (Table 1). Among them, metabolic pathway genes (E2.3.1.37, 5-aminolevulinate synthase and *Alox5*, arachidonate 5-lipoxygenase) and protein digestion and absorption-related genes (*cola1* and *cola2*, collagen type I alpha 1/2 chain) were upregulated in females, whereas the steroid hormone biosynthesis-related genes *hsd17b3* (hydroxysteroid 17-beta dehydrogenase 3) and *srd5a1* (3-oxo-5-alpha-steroid 4-dehydrogenase 1) were downregulated in females (Figure 5A). In the gonads of diploid females and males, the DEGs related to growth were mainly enriched in the cell cycle and DNA replication pathways. Most of the genes involved in the cell cycle (such as *cdc45*, *ccne*, *apc*, *orc1*, and *p53*) and DNA replication (*pcna*, *ssb*, *mcm2*, and *RNase-HI*) were expressed at higher levels in females than in males. The DEGs between diploid females and males in the liver were not significantly enriched in any growth-related pathways, but were mainly enriched in pathogenic infection and immune-related pathways, whereas those in the muscle were mainly enriched in glycolysis/gluconeogenesis and glucagon signaling pathways. These pathways-related genes (*gpi*, *pgk*, *pgam*, *eno*, and *gapdh*) were all upregulated in females (Figure 5B and Figure 6A). In the comparison of tetraploid females and males (T-B/G/L/M-F vs. T-B/G/L/M-M), the DEGs involved in protein digestion and absorption and drug metabolism-cytochrome P450 pathways enriched in the brain were upregulated in females, while those involved in cardiac muscle contraction, insulin secretion, MAPK signaling pathway, and calcium signaling pathway were downregulated. Interestingly, in gonads, the mRNA expression levels of ribosome biogenesis in eukaryotes and protein processing in endoplasmic reticulum process-related genes (*utp22*, *utp6*, *emg1*, *ssr1*, *eif2α*, and *ire1*) were also upregulated in females. In the liver, the expression levels of the enriched ribosome-related genes (*lp1*, *l11e*, and *l23e*) and glycolysis/gluconeogenesis-related genes (*pfk* and *pgk*) were upregulated in females, while the biosynthesis of unsaturated fatty acids pathway gene *scd* and steroid hormone biosynthesis-related genes *cyp7a1* and *hsd11b2* were downregulated. In the muscle, the expression of the DEGs involved in tight junction (*myl6*, *myh9*, and *myl12*) and fatty acid degradation (*cpt1*) processes were higher in males than in females. However, the expression of genes related to glycolysis and linoleic acid metabolism was reversed, whereby it was higher in females than in males.

In addition, in order to reveal the pathways regulating polyploid growth dimorphism, we selected the growth-related pathways enriched by the DEGs in T-B/G/L/M-F vs. D-B/G/L/M-F and T-B/G/L/M-M vs. D-B/G/L/M-M for analysis. According to the results of KEGG pathway enrichment analysis, most of the DEGs identified in the four tissues in the comparison of tetraploid and diploid females (T-B/G/L/M-F vs. D-B/G/L/M-F) were significantly enriched in the oxidative phosphorylation pathway (Table 1). Most of the genes (NADH-ubiquinone oxidoreductase chain 3 (*nd3*), *nd4*, *nd4l*, *cytb*, cytochrome c oxidase subunit 3 (*cox3*), *cox4*, and *cox5*) exhibited higher expression in tetraploids than in diploids (Figure 5C and Figure 6B), whereas the expression of the DEGs related to steroid hormone biosynthesis and fatty acid degradation pathways was generally lower in tetraploids than in diploids. Similarly, in the comparison of tetraploid and diploid males (T-B/G/L/M-M vs. D-B/G/L/M-M) in multiple tissues, DEGs that were relatively highly expressed in tetraploid males were also significantly enriched in oxidative phosphorylation and glycolysis pathways, while those with lower expression in tetraploid males were mainly enriched in steroid hormone synthesis (Table 1). A heat-map was constructed by filtering some key genes related to glycolysis and oxidative phosphorylation pathways, and the results showed that genes related to glycolysis had high expression levels in both diploid and tetraploid muscle tissues, while most of the genes related to oxidative phosphorylation had high expression levels in multiple tissues (Figure 6).

### 3.6. Verification of RNA-seq Data by qPCR

**Figure 7:**
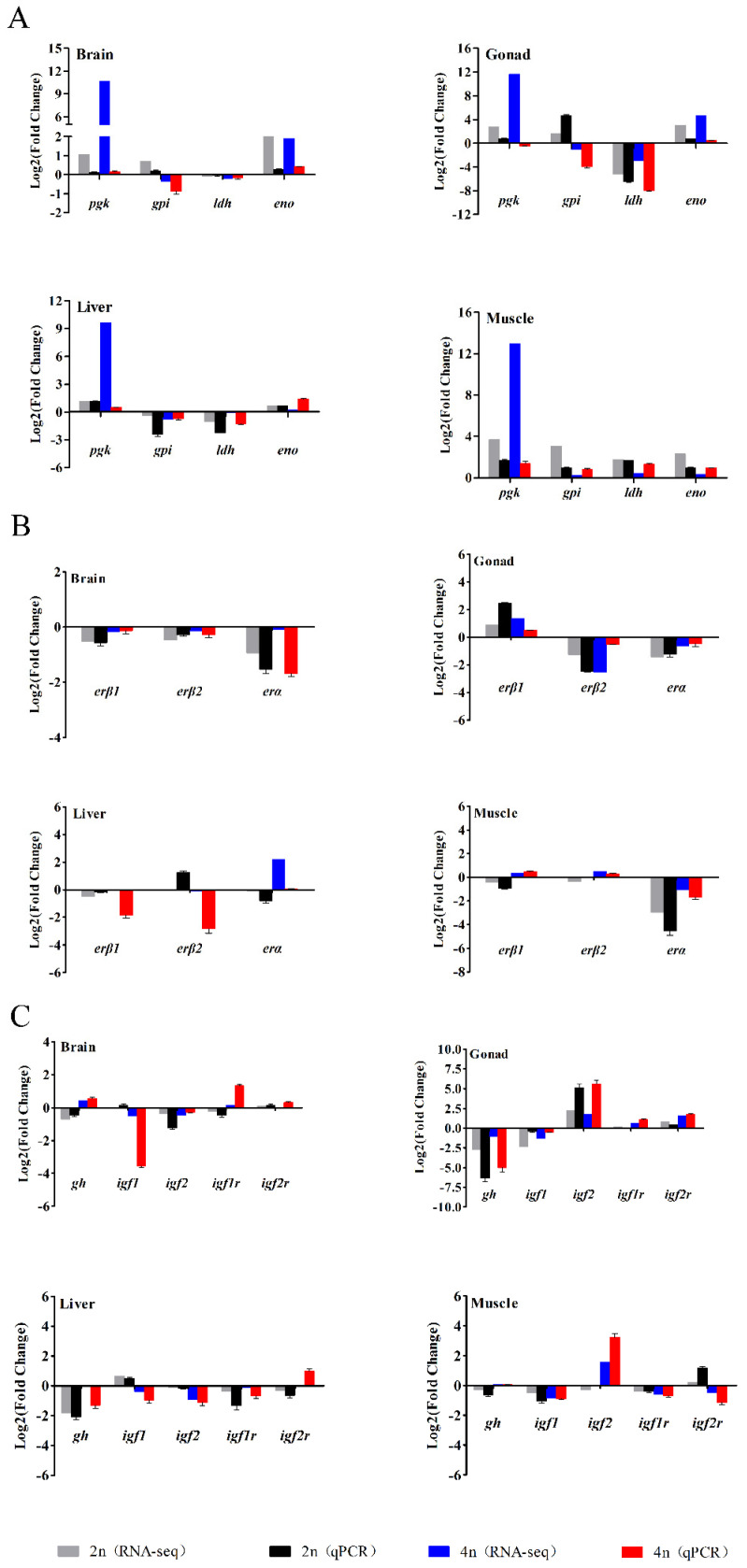
Comparison of the expression profiles of genes involved in the glycolytic pathway. (**A**) Three estrogen receptor genes (**B**) and several reported growth-related genes (**C**) determined by RNA-seq and qPCR in four tissues of female loaches. Each bar represents the expression fold change in a gene compared to that in the male loaches.

To confirm the reliability of the RNA-seq data, four genes (*pgk*, *gpi*, *ldh*, and *eno*) involved in the glycolytic pathway, three estrogen receptor genes (*erα*, *erβ1*, and *erβ2*) and several growth-related genes (*gh*, *igf1*, *igf2*, *igf1r*, and *igf2r*) were selected for qPCR analysis. The expression levels of these genes are shown in Figure 7. Overall, the differential expression patterns detected by the two methods showed good consistency, although the exact Log2 (fold-change) in the genes at several data points varied between the RNA-seq and qPCR analyses.

## 4. Discussion

Growth is one of the most important economic traits for aquaculture fish, and is of great significance to the development of the aquaculture industry [39]. Understanding the molecular mechanisms of sexual and polyploid growth dimorphisms will provide a theoretical basis for the cultivation of fast-growing populations with stable and uniform growth performance in aquaculture. So far, research on the growth differences between female and male fish has mainly focused on the aspects of feeding and digestion [5], growth, reproductive energy allocation [40], inheritance, genotypes and phenotypes [41], steroid hormone levels [42], and growth axis genes [43,44]. Moreover, past research on polyploid growth dimorphism has focused on the expression of a single gene [18]. Unlike most previous studies, we performed comparative transcriptome integration analysis of multiple tissues of diploids and tetraploids in both sexes. This is the first report of a combined analysis of sexual and polyploid growth dimorphisms in fish, and it will help us understand the regulatory mechanisms involved and provide basic information for breeding the loach strains with fast growth and uniform size.

In the comparison of male and female loach transcriptomes, our results demonstrated that the main pathways related to growth differences between diploid females and males were glycolysis, hormone synthesis, and cell cycle. Specifically, the expression levels of unigenes related to metabolism (*gpi*, *pgk*, *pgam*, etc.) and cell cycle (*cdc45*, *cyce*, *apc*, etc.) were higher in females than in males, while the expression level of hormone synthesis-related genes (such as *hsd17b3*) in females was lower than that in males. Similar to the male and female diploid comparison group, the male and female tetraploid comparison group was also significantly enriched in glycolysis/gluconeogenesis, and the glycolysis/gluconeogenesis level was also higher in females than in males. Glycolysis/gluconeogenesis is the central pathway of most organisms, providing energy in the form of ATP and reducing power, pyruvate to fuel for the tricarboxylic acid cycle, and precursors for secondary metabolism, amino acid and fatty acid biosynthesis [45]. PGK is a key enzyme for adenosine triphosphate (ATP) generation in glycolysis. Taken together, our results showed that female individuals had higher metabolic levels than male individuals, but lower levels of steroid hormone synthesis than males. On theoretical grounds, there is a link between growth rate and metabolic rate, with fast growth requiring fast metabolism to provide the growing organism with the necessary materials and energy [46]. Glycolysis has also been found upregulated in the muscle of domestic rainbow trout compared to their wild counterparts [47], and also in faster-growing fish of the same species; this up-regulation has been associated with an increased muscle energy demand in fast-growing fish [48,49]. Studies in humans have also shown that men grow faster than women and have higher metabolic levels [50]. However, in cows where males grow faster than females, the total glucose metabolism is two-fold higher in males than in females, but the activity of the pentose phosphate pathway is four times higher in females than in males [51]. The molecular mechanisms underlying these effects are unclear. The earlier view is that females may have increased metabolic levels to reduce the effects of oxygen free radicals, allowing them to grow faster and have higher viability [52]. In our study, we speculate that the higher metabolic levels of female individuals provide more energy for growth, and the reduction in hormone synthesis levels also saves energy. Therefore, female individuals grow faster than male individuals and are larger in size.

In addition, protein digestion and absorption-related genes were also significantly upregulated in females, among which *cola1* and *cola2* were the most enriched. COLA1 and COLA2 are members of the type I collagen family. Collagen is not only an important protein in animal connective tissue, but also the main component of the extracellular matrix (*ECM*), which can provide support for cell growth [53]. Other collagens have also been found to be closely related to the development of osteogenic cell [54,55]. Our results indicated that the expression level of type I collagen is higher in females with faster growth than in males with slower growth, which is consistent with the reported growth-promoting effect of collagens. However, among the DEGs, the most downregulated genes in females were *cacna1c* and *cacna1d*, which were involved in multiple pathways, including insulin secretion, calcium signaling pathway and MAPK signaling pathway. It has been reported that mice with *cacna1c* and *cacna1d* deficiencies often lack of exercise and social interaction skills [56]. Female individuals may reduce exercise and social skills by regulating the decreased expression of *cacna1c* and *cacna1d* in the body, thus reducing energy expenditure and maintaining a larger body size.

In the comparative analysis of brain, liver, muscle, and gonad tissues of male and female individuals, both DEGs in male and female brains of diploids and tetraploids were enriched in metabolic processes, such as amino acid metabolism, glycolysis/gluconeogenesis, and oxidative phosphorylation. The DEGs in the muscle were also significantly enriched in metabolic processes, whereas those in the liver were mainly related to *Staphylococcus aureus* infection. Growth is regulated by the growth axis “hypothalamic-pituitary-target organ” [57]. DEGs in both male and female brains and muscles were enriched in the same processes, suggesting that the hypothalamus mainly acts on the target organ of muscle to regulate the growth differences between male and female loaches. It is not surprising that the DEGs in the liver are enriched in immune-related processes, as the liver is an important immune organ [58]. Although gonads can regulate growth, they mainly play a role in reproduction.

The transcriptome integration analysis results of tetraploids and diploids showed that regardless of sex, oxidative phosphorylation was the most significantly enriched in multiple tissues. Genes related to this pathway (such as *cox3*, *nd4*, *nd4l*, *cytb*, *qcr2*, and *qcr10*) were upregulated in tetraploids. In Wistar rats and monkeys, where males have a growth advantage, the levels of oxidative phosphorylation were higher in females than in males [59,60]. Our results showed that oxidative phosphorylation levels were higher in fast-growing individuals, which is contrary to the results in mammals. The level of oxidative phosphorylation reflects the mitochondrial activity [61]. Oxidative phosphorylation provides most of the ATP for animals and plants to maintain homeostasis in life and energy metabolism [62]. Moreover, oxidative phosphorylation is an important form of energy metabolism. This suggests that the difference in growth of tetraploids and diploids is closely related to energy metabolism. Our results indicate that the mechanisms regulating energy metabolism and growth in fish may be different from those in mammals. Studies have shown that estrogen is involved in the regulation of mitochondrial activity in animals, including regulation of mitochondrial biogenesis, oxygen consumption, and energy production [63,64]. Estrogen receptor is the key to the physiological role of estrogen. There are two subtypes of estrogen receptor genes (*erα* and *erβ*) in mammals, while three subtypes of estrogen receptor genes (*erα*, *erβ1*, and *erβ2*) in teleost fish [65,66]. Therefore, we speculate that the different estrogen receptor gene types between mammals and fish may result in different ways in which they regulated energy metabolism.

In general, regardless of sexual or polyploidy growth dimorphisms, processes related to growth differences were significantly enriched in pathways related to energy metabolism, including glycolysis/gluconeogenesis, oxidative phosphorylation, hormone synthesis, and fat metabolism. Among them, glycolysis/gluconeogenesis and oxidative phosphorylation pathways were upregulated in fast-growing loach individuals (females, tetraploids), while hormone synthesis and fat metabolism pathways were upregulated in slow-growing loach individuals (males, diploids). Studies have demonstrated that glucose oxidative metabolism reduces the formation of free radicals, while fat metabolism promotes the formation of free radicals [67]. Free radicals play an important role in the entire process of life activities. Thus, it is suggested that the reduction of free radicals weakens the activity of the entire body and provides more energy for growth, while the increase in free radicals accelerates the body’s activities and increases energy consumption, leaving less energy for growth. Further research is needed to be undertaken to explore the molecular mechanisms by which females and males regulate energy metabolism balance and growth in fish.

## 5. Conclusions

In conclusion, by using comparative transcriptome analysis, we successfully identified the DEGs related to sexual and polyploid growth dimorphisms in *M. anguillicaudatus*. Based on transcriptome integration analysis, we found that regardless of the comparison of sexes or ploidy, the DEGs were mainly involved in glycolysis/gluconeogenesis, oxidative phosphorylation, steroid hormone biosynthesis, fatty acid degradation, and elongation. Our results suggest that the differences in energy metabolism levels, steroid hormone synthesis, and fatty acid degradation abilities might be important reasons for the sexual and polyploidy dimorphisms in loaches. Specifically, fast-growing loaches (tetraploids, females) have higher levels of energy metabolism and lower steroid hormone synthesis and fatty acid degradation abilities than slow-growing loaches (diploids, males). Our study not only indicates the direction for us to further investigate the molecular mechanisms of sexual and polyploidy growth dimorphisms of loaches, but also provides essential gene information for future functional studies.

## Figures and Tables

**Table 1:** Growth-related KEGG pathways in each comparison group.

Group	KEGG Terms	Upregulated Genes	Downregulated Genes	Corrected *P*-Value
D-B-F vs. D-B-M	Porphyrin and chlorophyll metabolism	E2.3.1.37(5-aminolevulinate synthase)		4.36 × 10^−2^
	Glycine, serine and threonine metabolism	E2.3.1.37(5-aminolevulinate synthase)		4.36 × 10^−2^
	Arachidonic acid metabolism	*alox5*		4.36 × 10^−2^
	Protein digestion and absorption	*cola1*, *cola2*		8.82 × 10^−2^
	Ovarian steroidogenesis	*alox5*		4.36 × 10^−2^
	Steroid hormone biosynthesis		*hsd17b3*, *srd5a1*	4.36 × 10^−2^
D-G-F vs. D-G-M	Cell cycle	*cdc45*, *ccne*, *apc1*, *orc1*, *p53*, *mcm2*		4.63 × 10^−2^
	Spliceosome	*cdc5*, *cype*, *prp17*, *prp18*, *eif4a1*		7.83 × 10^−2^
	DNA replication	*pcna*, *ssb*, *RNaseHI*, *mcm2*, *mcm4*		8.47 × 10^−2^
D-L-F vs. D-L-M	Taurine and hypotaurine metabolism	*csad*		3.39 × 10^−1^
	Arginine and proline metabolism	*prodh2*	*aoc1*	3.39 × 10^−1^
D-M-F vs. D-M-M	Glycolysis/gluconeogenesis	*gpi*, *pgk*, *pgam*, *ldh*		2.58 × 10^−7^
	Glucagon signaling pathway	*pgam*, *ldh*		9.64 × 10^−3^
	Starch and sucrose metabolism	*gpi*		2.30 × 10^−2^
T-B-F vs. T-B-M	Glycolysis/gluconeogenesis	*pgk*		4.88 × 10^−1^
	Protein digestion and absorption	*col1a*, *cola2*		3.31 × 10^−1^
	Drug metabolism-cytochrome P450	*fmo*		3.54 × 10^−1^
	Taurine and hypotaurine metabolism	*ggt1-5*		4.88 × 10^−1^
	Cardiac muscle contraction		*cacna1c*, *cacna1d*	4.94 × 10^−1^
	GnRH signaling pathway		*cacna1c*, *cacna1f*	4.88 × 10^−1^
	Insulin secretion		*cacna1c*	4.88 × 10^−1^
	MAPK signaling pathway		*cacna1a*	5.77 × 10^−1^
	Calcium signaling pathway		*cacna1c*	5.77 × 10^-1^
T-G-F vs. T-G-M	Ribosome biogenesis in eukaryotes	*utp22*, *utp6*, *imp3*, *ck2a*, *emg1*		2.77 × 10^−1^
	Protein processing in endoplasmic reticulum	*atf6*, *ire1*, *eif2ak1*, *eif2α*, *ssr1*		3.17 × 10^−1^
T-L-F vs. T-L-M	Ribosome	*lp1*, *lp2*, *l11e*, *l23e*		4.92 × 10^−11^
	Protein processing in endoplasmic reticulum	*ssr1*	*hsp70*	8.01 × 10^−2^
	Glycolysis/gluconeogenesis	*pfka*, *pgk*		2.93 × 10^−1^
	Biosynthesis of unsaturated fatty acids		△*9-desaturase*	2.26 × 10^−2^
	Steroid hormone biosynthesis		*cyp7a1*, *hsd11b2*	7.18 × 10^−2^
T-M-F vs. T-M-M	Tight junction		*myl6*, *myl12*, *myh9*	3.85 × 10^−2^
	DNA replication	*rfc3/5*		1.98 × 10^−1^
	Linoleic acid metabolism	*cyp2j*		1.98 × 10^−1^
	Fatty acid degradation		*cpt1*	2.16 × 10^−1^
T-B-F vs. D-B-F	Oxidative phosphorylation	*cox3*, *cox5b*, *cytb*, *nd3*, *nd4*	*cox1*, *atpef0a*	1.04 × 10^−4^
	Cell cycle	*pcna*, *cyca*, *cycb*, *apc/c*		4.07 × 10^−1^
	PI3K-Akt signaling pathway	*creb*, *akt*, *pp2a*, *ras*	*itga*, *pkc*, *bim*	7.58 × 10^−1^
	PPAR signaling pathway	*lpl*, *acs*, *scp*		8.70 × 10^−1^
	Protein digestion and absorption	*slc15a1*, *mme*		1.20 × 10^−1^
	Regulation of actin cytoskeleton	*actb-g1*, *myl2*, *myl5*	*egfr*, *fgfr2*, *fgfr3*	9.99 × 10^−1^
	Drug metabolism-cytochrome P450	*mao*, *ugt*, *gst*		6.32 × 10^−1^
	GnRH signaling pathway	*slc5a6*, *rft2*	*cubn*, *abcc1*	9.99 × 10^−1^
	Estrogen signaling pathway	*gper*, *mmp*, *creb1*		7.58× 10^−1^
	Fatty acid degradation		*mecr*, *fada*, *acaa2*	9.99 × 10^−1^
	Fatty acid elongation		*elovl1*, *mecr*, *acaa2*, *ter*	8.70 × 10^−1^
	Cardiac muscle contraction	*myh6/7*, *myl2*, *myl3*, *atp1a*, *atp1b*		3.82 × 10^−1^
T-G-F vs. D-G-F	Oxidative phosphorylation	*cox3*, *nd4*, *nd4l*, *cytb*	*cox1*, *sdhb*	6.35 × 10^−1^
	Arachidonic acid metabolism	*prxl2b*, *cbr1*, *cbr2*, *ptgds*, *cyp4f*, *alox12*		6.35 × 10^−1^
	p53 signaling pathway	*igf*, *tsp*, *mdm2*, *cyclinb*, *casp8*, *perp*		6.35 × 10^−1^
	Ascorbate and aldarate metabolism			6.35 × 10^−1^
T-L-F vs. D-L-F	Oxidative phosphorylation	*cox3*, *nd4*, *nd4l*, *cytb*	*cox1*	1.77 × 10^−6^
	Mineral absorption	*zip4*, *atpase*		1.04 × 10^−5^
	Vitamin digestion and absorption	*apob-48*, *lrat*, *rft2*		1.64 × 10^−4^
T-M-F vs. D-M-F	Tight junction	*actin*	*myl6, myl1, myh9*	5.02 × 10^−5^
	Cardiac muscle contraction	*actin*, *tpm1*		4.64 × 10^−13^
	Oxidative phosphorylation	*cox3*, *nd4*, *nd4l*, *cytb*, *qcr2*, *qcr10*	*cox1*	1.00 × 10^−9^
	Protein digestion and absorption	*slc8a*, *mme*	*slc3a2*	6.82 × 10^−5^
T-B-M vs. D-B-M	Oxidative phosphorylation	*cox3*, *cox5*, *cytb*, *nd3*, *nd4*	*cox1*, *atpef0a*	2.30 × 10^−2^
	Glutathione metabolism	*gpx*, *anpep*, *rrm1*	*gpx4*, *ggct*	1.45 × 10^−2^
	Steroid hormone biosynthesis		*cyp11b1*, *cyp11b2*, *srd5a1*	1.88 × 10^−1^
T-G-M vs. D-G-M	Cell cycle	*cdc45*, *ccne*, *apc1*, *orc1*, *p53*, *mcm2*		3.50 × 10^−6^
	DNA replication	*pcna*, *mcm2*, *mcm4*		4.25 × 10^−7^
	Regulation of actin cytoskeleton	*iqgap*, *fak*, *rho*, *pak1*, *f-actin*, *drf3*		1.04 × 10^−1^
T-L-M vs. D-L-M	Mineral absorption	*mt*, *fpn1*	*hmox*, *znt1*	1.51 × 10^−3^
	Cardiac muscle contraction	*oplah*, *gpx*, *ggt*	*ggct*, *g6pd*	5.45 × 10^−1^
	Steroid hormone biosynthesis		*cyp2r1*, *cyp51*, *dwf*, *fdft1*	1.18 × 10^−6^
	Fat digestion and absorption	*abca1*, *apoa*, *dgat*		2.44 × 10^−3^
	Oxidative phosphorylation	*cox3*, *cox5*, *cytb*, *nd3*, *nd4*		1.40 × 10^−2^
	Glycolysis/gluconeogenesis	*fbp*, *tpi*, *eno*, *adh1*, *g6pc*		1.93 × 10^−1^
T-M-M vs. D-M-M	Glycolysis/gluconeogenesis	*gpi*, *pgk*, *pgam*, *ldh*, *tpi*		6.20 × 10^−7^
	Tight junction	*actin*, *lgl1*	*claudin*, *pp2a*, *myl2*	1.00 × 10^−9^
	Cardiac muscle contraction	*actin*, *tpm*, *atp*	*tnt*, *tnc*	7.34 × 10^−14^
	Oxidative phosphorylation	*cox3*, *nd4*, *nd4l*, *cytb*, *qcr2*, *qcr10*	*cox1*	7.34 × 10^−14^

Note: brain, gonad, liver, and muscle samples from diploid females were compared with those from diploid males, abbreviated as D-B-F vs. D-B-M, D-G-F vs. D-G-M, D-L-F vs. D-L-M, and D-M-F vs. D-M-M, respectively, and T-B-F vs. T-B-M, T-G-F vs. T-G-M, T-L-F vs. T-L-M, and T-M-F vs. T-M-M represent the comparisons of those samples between females and males in tetraploids. T-B-F vs. D-B-F, T-G-F vs. D-G-F, T-L-F vs. D-L-F, and T-M-F vs. D-M-F represent the comparison of brain, gonad, liver, and muscle samples of tetraploid and diploid females, respectively, and T-B-M vs. D-B-M, T-G-M vs. D-G-M, T-L-M vs. D-L-M, and T-M-M vs. D-M-M represent comparisons between tetraploid and diploid males.

## Data Availability

All sequencing data have been uploaded to the Sequence Read Archive (SRA) of the National Center for Biotechnology Information (NCBI accession number PRJNA678824).

## Supplementary Materials

The following are available online at https://www.mdpi.com/article/10.3390/biology10090935/s1, Table S1: Primers sequences used in qPCR experiments, Table S2: Information and quality of RNA-seq. Table S3: Assembly statistics of the transcriptome data, Table S4: Assembly length distribution, Table S5: Top six enriched GO terms for the DEGs, Table S6: Significantly enriched GO annotations for DEGs between diploid female and male gonads, Table S7: Top six enriched KEGG terms for the DEGs, Table S8: Significantly enriched GO annotations for DEGs between tetraploid female and male gonads, Table S9: KEGG enrichment results of common DEGs in gonads, Figure S1: DNA content of diploid and tetraploid cells of loaches measured by flow cytometry. (A), *Paramisgurnus dabryanus*, a known diploid, was used as a control. (B), diploid *M*. *anguillicaudatus*, which have the same DNA content as the diploid control; (C), tetraploid *M*. *anguillicaudatus*, which have doubled DNA content compared with diploids.

## References

[1] Mei J., Gui J. (2015). Genetic basis and biotechnological manipulation of sexual dimorphism and sex determination in fish. Sci. China Life Sci..

[2] Connallon T., Knowles L. (2005). Intergenomic conflict revealed by patterns of sex-biased gene expression. Trends Genet..

[3] Rinn J., Snyder M. (2005). Sexual dimorphism in mammalian gene expression. Trends Genet..

[4] Ji X. (2009). Artificial Gynogenesis, Genetic Analysis and Differential Expression of Growth-Related Genes in Half-Smooth Tongue Sole.

[5] Wu B. (2013). Difference of Growth Performance, Digestive Enzyme Activities and Growth Hormone (GH) Expressions between Male and Female Scatophagus argus Linnaeus.

[6] Cutting A., Chue J., Smith C. (2013). Just how conserved is vertebrate sex determination?. Dev. Dyn..

[7] Wang B., Guo G., Wang C., Lin Y., Wang X., Zhao M., Guo Y., He M., Zhang Y., Pan L. (2010). Survey of the transcriptome of *Aspergillus oryzae* via massively parallel mRNA sequencing. Nucleic Acids Res..

[8] Li Y., Wang G., Tian J., Liu H., Yang H., Yi Y., Wang J., Shi X., Jiang F., Yao B. (2012). Transcriptome analysis of the silkworm (*Bombyx mori*) by high-throughput RNA sequencing. PLoS ONE.

[9] Zhang Z., Wang Y., Wang S., Liu J., Warren W., Mitreva M., Walter R. (2011). Transcriptome analysis of female and male *Xiphophorus maculatus* Jp 163 A. PLoS ONE.

[10] Salem M., Rexroad C., Wang J., Thorgaard G., Yao J. (2010). Characterization of the rainbow trout transcriptome using Sanger and 454-pyrosequencing approaches. BMC Genom..

[11] Tao W., Yuan J., Zhou L., Sun L., Sun Y., Yang S., Li M., Zeng S., Huang B., Wang D. (2013). Characterization of gonadal transcriptomes from nile tilapia (*Oreochromis niloticus*) reveals differentially expressed genes. PLoS ONE.

[12] Sun L., Wang C., Huang L., Wu M., Zuo Z. (2012). Transcriptome analysis of male and female *Sebastiscus marmoratus*. PLoS ONE.

[13] Sun F., Liu S., Gao X., Jiang Y., Perera D., Wang X., Li C., Sun L., Zhang J., Kaltenboeck L. (2013). Male-biased genes in catfish as revealed by RNA-seq analysis of the testis transcriptome. PLoS ONE.

[14] Ribas L., Pardo B., Fernandez C., Antonio Alvarez-Dios J., Gomez-Tato A., Isabel Quiroga M., Planas J., Sitja-Bobadilla A., Martinez P., Piferrer F. (2013). A combined strategy involving Sanger and 454 pyrosequencing increases genomic resources to aid in the management of reproduction, disease control and genetic selection in the turbot (*Scophthalmus maximus*). BMC Genom..

[15] Krysanov E., Golubtsov A. (2014). Karyotypes of four fish species from the Nile and Omo-Turkana basins in Ethiopia. J. Ichthyol..

[16] Comber S., Smith C. (2004). Polyploidy in fishes: Patterns and processes. Biol. J. Linn. Soc..

[17] Xiao J., Zou T., Chen Y., Chen L., Liu S., Tao M., Zhang C., Zhao R., Zhou Y., Long Y. (2011). Coexistence of diploid, triploid and tetraploid crucian carp (*Carassius auratus)* in natural waters. BMC Genet..

[18] Tao M., Liu S., Long Y., Zeng C., Liu J., Liu L., Zhang C., Duan W., Liu Y. (2008). The cloning of Dmc1 cDNAs and a comparative study of its expression in different ploidy cyprinid fishes. Sci. China Ser. C Life Sci..

[19] Zhou H., Ma T.Y., Zhang R., Xu Q., Shen F., Qin Y., Xu W., Wang Y., Li Y. (2016). Analysis of different ploidy and parent-offspring genomic DNA methylation in the loach *Misgurnus anguillicaudatus*. Int. J. Mol. Sci..

[20] Zhong H., Yi Z., Liu S., Tao M., Long Y., Liu Z., Zhang C., Duan W., Hu J., Song C. (2012). Elevated expressions of GH/IGF axis genes in triploid crucian carp. Gen Comp. Endocr..

[21] Tao M., Liu S., Zhan Z., Chen J., Liu W., Liu Y. (2014). Molecular cloning and comparative expression patterns of cyp19a1a of gene in different ploidy cyprinid fishes. J. Fish. China.

[22] Meirmans P., Liu S., Van Tienderen P. (2018). The Analysis of Polyploid Genetic Data. J. Hered..

[23] Odei D., Hagen O., Peruzzi S., Falk-Petersen I., Fernandes J. (2020). Transcriptome sequencing and histology reveal dosage compensation in the liver of triploid pre-smolt Atlantic salmon. Sci. Rep..

[24] Zhou R., Wu Y., Tao M., Zhang C., Liu S. (2015). MicroRNA profiles reveal female allotetraploid hybrid fertility. BMC Genet..

[25] Michaeloudes C., Kuo C., Haji G., Finch D., Halayko A., Kirkham P., Chung K., Adcock I. (2017). Metabolic re-patterning in COPD airway smooth muscle cells. Eur. Respir. J..

[26] Roosterman D., Meyerhof W., Cottrell G. (2018). Proton Transport Chains in Glucose Metabolism: Mind the Proton. Front. Neurosci..

[27] Tixier V., Bataillé L., Etard C., Jagla T., Weger M., Daponte J., Strähle U., Dickmeis T., Jagla K. (2013). Glycolysis supports embryonic muscle growth by promoting myoblast fusion. Proc. Natl. Acad. Sci. USA.

[28] Yuan Q., Miao J., Yang Q., Fang L., Fang Y., Ding H., Zhou Y., Jiang L., Dai C., Zen K. (2020). Role of pyruvate kinase M2-mediated metabolic reprogramming during podocyte differentiation. Cell Death Dis..

[29] Jiang D., LaGory E., Kenzelmann Brož D., Bieging K., Brady C., Link N., Abrams J., Giaccia A., Attardi L. (2015). Analysis of p53 transactivation domain mutants reveals Acad11 as a metabolic target important for p53 pro-survival function. Cell Rep..

[30] Lin T., Wu S. (2015). Reprogramming with Small Molecules instead of Exogenous Transcription Factors. Stem Cells Int..

[31] Dong C., Yuan T., Wu Y., Wang Y., Fan T., Miriyala S., Lin Y., Yao J., Shi J., Kang T. (2013). Loss of FBP1 by Snail-mediated repression provides metabolic advantages in basal-like breast cancer. Cancer Cell.

[32] Feng B., Yi S., Li R., Zhou X. (2017). Comparison of age and growth performance of diploid and tetraploid loach *Misgurnus anguillicaudatus* in the Yangtze River basin, China. Environ. Biol. Fish.

[33] Zhong J., Yi S., Ma L., Wang W. (2019). Evolution and phylogeography analysis of diploid and polyploid *Misgurnus anguillicaudatus* populations across China. Proc. Biol. Sci..

[34] Grabherr M., Haas B., Yassour M., Levin J., Thompson D., Amit I., Adiconis X., Fan L., Raychowdhury R., Zeng Q. (2011). Full-length transcriptome assembly from RNA-Seq data without a reference genome. Nat. Biotechnol..

[35] Trapnell C., Williams B., Pertea G., Mortazavi A., Kwan G., van Baren M., Salzberg S., Wold B., Pachter L. (2010). Transcript assembly and quantification by RNA-seq reveals unannotated transcripts and isoform switching during cell differentiation. Nat. Biotechnol..

[36] Love M., Huber W., Anders S. (2014). Moderated estimation of fold change and dispersion for RNA-seq data with DESeq2. Genome Biol..

[37] Kanehisa M., Araki M., Goto S., Hattori M., Hirakawa M., Itoh M., Katayama T., Kawashima S., Okuda S., Tokimatsu T. (2008). KEGG for linking genomes to life and the environment. Nucleic Acids Res..

[38] Livak K., Schmittgen T. (2001). Analysis of Relative Gene Expression Data Using Real-Time Quantitative PCR and the 2^−ΔΔCT^ Method. Methods.

[39] Blay C., Haffray P., Bugeon J., D’Ambrosio J., Dechamp N., Collewet G., Enez F., Petit V., Cousin X., Corraze G. (2021). Genetic parameters and genome-wide association studies of quality traits characterised using imaging technologies in rainbow trout, *Oncorhynchus mykiss*. Front. Genet..

[40] Wang X., Li C., Xie Z., Fan W., Zhang J. (2006). Studies on the growth difference of the male and female *Siniperca chuatsi*. Freshw. Fish..

[41] Toguyeni A., Fauconneau B., Fostier A., Abucay J., Mair G., Baroiller J. (2002). Influence of sexual phenotype and genotype, and sex ratio on growth performances in tilapia, *Oreochromis niloticus*. Aquaculture.

[42] Zhang J., Ma W., He Y., Wu J., Dawar F., Ren F., Zhao X., Mei J. (2016). Sex biased expression of ghrelin and GHSR associated with sexual size dimorphism in yellow catfish. Gene.

[43] Chatchaiphan S., Srisapoome P., Kim J., Devlin R., Na-Nakorn U. (2017). De novo transcriptome characterization and growth-related gene expression profiling of diploid and triploid bighead catfish (*Clarias macrocephalus Gunther*, 1864). Mar. Biotechnol..

[44] Ma W., Wu J., Zhang J., He Y., Gui J., Mei J. (2016). Sex differences in the expression of GH/IGF axis genes underlie sexual size dimorphism in the yellow catfish (*Pelteobagrus fulvidraco*). Sci. China-Life Sci..

[45] Plaxton W. (1996). The organization and regulation of plant glycolysis. Annu. Rev. Plant Physiol..

[46] Montes L., Le Roy N., Perret M., de Buffrenil V., Castanet J., Cubo J. (2007). Relationships between bone growth rate, body mass and resting metabolic rate in growing amniotes: A phylogenetic approach. Biol. J. Linn. Soc..

[47] Tymchuk W., Sakhrani D., Devlin R. (2009). Domestication causes large-scale effects on gene expression in rainbow trout: Analysis of muscle, liver and brain transcriptomes. Gen. Comp. Endocrinol..

[48] Danzmann R., Kocmarek A., Norman J., Rexroad C., Palti Y. (2016). Transcriptome profiling in fast versus slow-growing rainbow trout across seasonal gradients. BMC Genom..

[49] Robledo D., Rubiolo J., Cabaleiro S., Martínez P., Bouza C. (2017). Differential gene expression and SNP association between fast- and slow-growing turbot (*Scophthalmus maximus*). Sci. Rep..

[50] Ray P., Conaghan J., Winston R., Handyside A. (1995). Increased number of cells and metabolic activity in male human preimplantation embryos following in vitro fertilization. J. Reprod. Fertil..

[51] Tiffin G., Rieger D., Betteridge K., Yadav B., King W. (1991). Glucose and glutamine metabolism in pre-attachment cattle embryos in relation to sex and stage of development. J. Reprod. Fertil..

[52] Rieger D. (1992). Relationships between energy metabolism and development of early mammalian embryos. Theriogenology.

[53] Sorushanova A., Delgado L., Wu Z., Shologu N., Kshirsagar A., Raghunath R., Mullen A., Bayon Y., Pandit A., Raghunath M. (2019). The Collagen Suprafamily: From Biosynthesis to Advanced Biomaterial Development. Adv. Mater..

[54] Izu Y., Ezura Y., Koch M., Birk D., Noda M. (2016). Collagens VI and XII form complexes mediating osteoblast interactions during osteogenesis. Cell Tissue Res..

[55] Wen Y., Yang H., Wu J., Wang A., Chen X., Hu S., Zhang Y., Bai D., Jin Z. (2019). COL4A2 in the tissue-specific extracellular matrix plays important role on osteogenic differentiation of periodontal ligament stem cells. Theranostics.

[56] Redecker T., Kisko T., Schwarting R., Wohr M. (2019). Effects of Cacna1c haploinsufficiency on social interaction behavior and 50-kHz ultrasonic vocalizations in adult female rats. Behav. Brain Res..

[57] Chen J., Cao M., Zhang A., Shi M., Tao B., Li Y., Wang Y., Zhu Z., Trudeau V.L., Hu W. (2018). Growth Hormone Overexpression Disrupts Reproductive Status Through Actions on Leptin. Front. Endocrinol..

[58] Wang B., Wangkahart E., Secombes C.J., Wang T. (2019). Insights into the Evolution of the Suppressors of Cytokine Signaling (SOCS) Gene Family in Vertebrates. Mol. Biol. Evol..

[59] Colom B., Oliver J., Roca P., Garcia-Palmer F. (2007). Caloric restriction and gender modulate cardiac muscle mitochondrial H2O2 production and oxidative damage. Cardiovasc. Res..

[60] Yan L., Ge H., Li H., Lieber S., Natividad F., Resuello R., Kim S., Akeju S., Sun A., Loo K. (2004). Gender-specific proteomic alterations in glycolytic and mitochondrial pathways in aging monkey hearts. J. Mol. Cell. Cardiol..

[61] Brand S., Ebner K., Mikoteit T., Lejri I., Gerber M., Beck J., Holsboer-Trachsler E., Eckert A. (2020). Influence of Regular Physical Activity on Mitochondrial Activity and Symptoms of Burnout-An Interventional Pilot Study. J. Clin. Med..

[62] Wilson D. (2017). Oxidative phosphorylation: Unique regulatory mechanism and role in metabolic homeostasis. J. Appl. Physiol..

[63] Klinge C. (2008). Estrogenic control of mitochondrial function and biogenesis. J. Cell. Biochem..

[64] Palmer B., Clegg D. (2015). The sexual dimorphism of obesity. Mol. Cell. Endocrinol..

[65] Meng X., Bartholomew C., Craft J. (2010). Differential expression of vitellogenin and oestrogen receptor genes in the liver of zebrafish, Danio Rerio. Anal. Bioanal. Chem..

[66] Zhang Y., Wang H., Qin F., Liu S., Wu T., Li M., Xu P., Zhang X., Wang X., Hu G. (2012). Molecular characterization of estrogen receptor genes in loach Paramisgurnus dabryanus and their expression upon 17 alpha-ethinylestradiol exposure in juveniles. Gen. Comp. Endocr..

[67] Speijer D., Manjeri G., Szklarczyk R. (2014). How to deal with oxygen radicals stemming from mitochondrial fatty acid oxidation. Philos. Trans. R. Soc. Lond. B Biol. Sci..

