# Supplementary figures and images for: Comparative Transcriptome Analysis Revealed Genes Involved in Sexual and Polyploid Growth Dimorphisms in Loach (Misgurnus anguillicaudatus)
Source: Biology (Basel). 2021 Sep 18;10(9):935. doi: 10.3390/biology10090935 (PMC8468957; doi:10.3390/biology10090935)

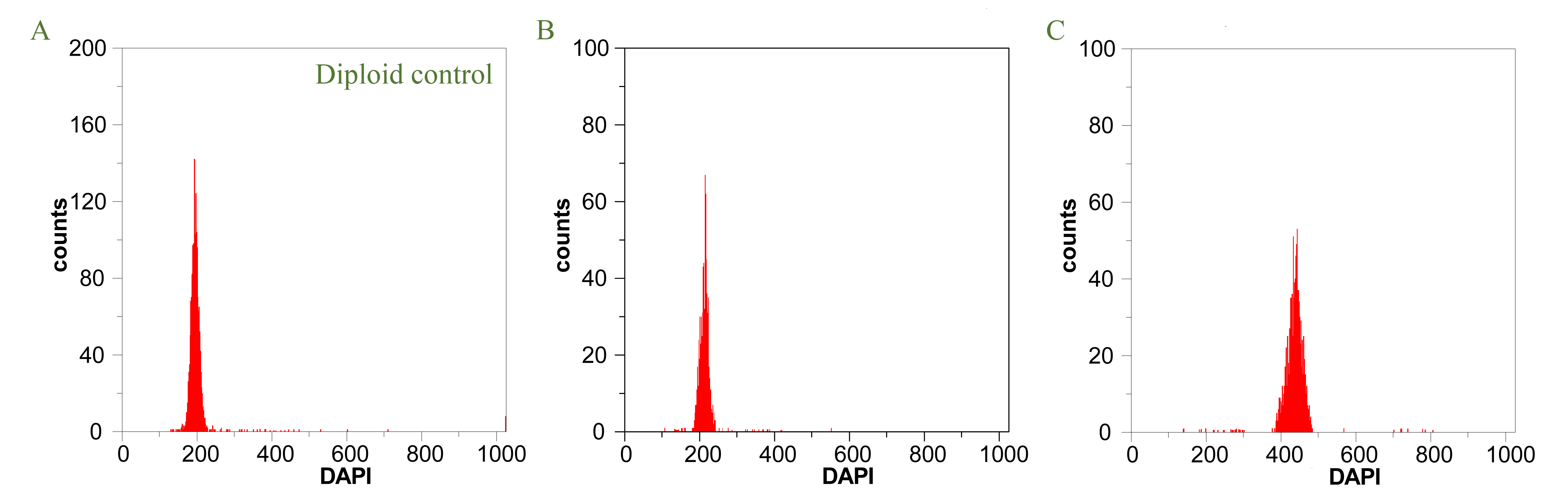

Supplement: Supplementary file 1 [file biology-10-00935-s001.zip › Supplementary Figure 1.tif]
